# Cross-clade CTL recognitions for clade B and A/E viruses in A/E virus-infected Japanese individuals

**DOI:** 10.1186/1742-4690-9-S2-P251

**Published:** 2012-09-13

**Authors:** K Watanabe, H Murakoshi, M Koyanagi, Y Tamura, R Maruyama, T Chikata, H Gatanaga, S Oka, M Takiguchi

**Affiliations:** 1 Kumamoto University, Tokyo, Japan; 2 AIDS Clinical Center, National Center for Global Health and Medicine, Tokyo, Japan

## Background

Cytotoxic T lymphocytes (CTLs) play an important role in the control of HIV-1. CTL responses to HIV-1 have been well studied in HIV-1 clade B-infected and clade C-infected individuals. However cross-clade CTL recognitions have not been well analyzed. In this study, we analyzed cross-clade CTL recognition for clade B and A/E viruses in A/E virus-infected Japanese individuals.

## Methods

PBMC samples were collected from chronically HIV-1 infected Japanese cohort in NCGM. Twenty-six clade A/E-infected individuals were analyzed by ELISPOT assay using the 11-mer overlapping peptides and then the responses of CTLs to these peptides was compared to those from 402 clade B-infected Japanese individuals. Thereafter CTL responses to each single peptide and to truncated peptides were evaluated by ELISPOT assay and intracellular cytokine staining (ICC) assay, respectively.

## Results

Similar level of CTL responses to Gag, Pol and Nef were found in clade A/E-infected individuals as compared to that in clade B-infected ones. We identified 15 cross-clade CTL epitopes from 14 cocktails where the frequency of responders was high in clade A/E infected samples. The sequences of 7 epitopes were conserved between clade B and clade A/E viruses, whereas 8 epitopes showed different amino acid sequences between two viruses. In these 8 epitope regions, we confirmed cross-clade CTL recognition by ICC assay using clade A/E consensus sequence peptide.

## Conclusion

Cross-clade CTLs were predominantly induced in clade A/E-infected individuals by clade B consensus sequence peptides in this study. Moreover, CTL responses were induced not only in conserved region but also in different sequence region between the 2 viruses, indicating that polymorphic sequence epitopes among clades can be also candidate for the target of CTL-based vaccines. Further analysis of cross-clade CTL recognition is needed for the widely applicable vaccine development.

